# Phenotype and genetic determination of resistance to common disinfectants among biofilm-producing and non-producing *Pseudomonas aeruginosa* strains from clinical specimens in Iran

**DOI:** 10.1186/s12866-022-02524-y

**Published:** 2022-05-07

**Authors:** Mehdi Bakht, Safar Ali Alizadeh, Sara Rahimi, Raana Kazemzadeh Anari, Mohammad Rostamani, Amir Javadi, Amir Peymani, Seyed Mahmoud Amin Marashi, Farhad Nikkhahi

**Affiliations:** 1 Medical Microbiology Research Center, Qazvin University of Medical Sciences, Qazvin, Iran; 2 Student Research Committee, Qazvin University of Medical Sciences, Qazvin, Iran; 3 Department of Community Medicine, Qazvin University of Medical Sciences, Qazvin, Iran

**Keywords:** Nosocomial infection, Disinfectant-resistance, Biofilm, Hospital disinfectants, *Pseudomonas aeruginosa*, Clinical isolates

## Abstract

**Background:**

*Pseudomonas aeruginosa* is a common pathogen in Hospitalized patients, and its various resistance mechanisms contribute to patient morbidity and mortality. The main aims of the present study were to assess the susceptibility of biofilm-producing and non-producing *P. aeruginosa* isolates to the five commonly used Hospital disinfectants, to evaluate the synergistic effect of selected disinfectants and Ethylene-diamine-tetra acetic acid (EDTA), and the effect of exposure to sub-inhibitory concentrations of Sodium hypochlorite on antimicrobial susceptibility test.

**Results:**

The results showed that sodium hypochlorite 5% and Ethanol 70% were the most and least effective disinfectants against *P. aeruginosa*, respectively. The addition of EDTA significantly increased the effectiveness of the selected disinfectants. The changes in the antibiotic-resistance profiles after exposure to sub-inhibitory concentrations of disinfectants were observed for different classes of antibiotics (Carbapenems, Aminoglycosides, Cephalosporins, Fluoroquinolones). As well as near the all isolates harbored efflux pump genes and 117 (97.5%) of isolates produced biofilm.

**Conclusion:**

In the current study, the mixture of disinfectant and EDTA were the most suitable selection to disinfect Hospital surfaces and instruments. Also, it was clear that exposure to sub-inhibitory concentrations of Sodium hypochlorite results in resistance to some antibiotics in *P. aeruginosa* species. Strong and intermediate biofilm formers belonged to MDR/XDR strains. Future studies should include more complex microbial communities residing in the Hospitals, and more disinfectants use in Hospitals.

## Background

*P. aeruginosa* is a gram-negative bacilli and is known as human opportunistic pathogen, especially for high-risk patients, including burn wounds, immunocompromised patients, and cystic fibrosis [1, 2]. It is a member of the ESKAPE (*Enterococcus faecium*, *Staphylococcus aureus*, *Klebsiella pneumoniae*, *Acinetobacter baumannii*, *P. aeruginosa*, and *Enterobacter* species) the leading cause of nosocomial infections throughout the world [3]. Also, *P. aeruginosa* is one of the bacteria that the World Health Organization (WHO) named as antibiotic‐resistant “priority pathogens” and has acquired and expanded different kinds of resistance mechanisms [3–5]. *P. aeruginosa* infections are difficult to treat because of their ability to acquire resistance to antibiotics [6]. *P. aeruginosa* possesses both cell-associated (lipopolysaccharide, flagella, alginate/biofilm pili, lectins) and extracellular (cytotoxin, proteases, hemolysins, siderophores, pyocyanin, exoenzyme S, exotoxin A, exoenzyme U, etc.) virulence factors [7]. Multi-drug-resistant (MDR) or Extensively drug-resistant (XDR) strains, in the nosocomial base, are a global threat to health care systems and vulnerable patients, and they have been reported to cause a large number of Hospital incidence in high-risk patients such as patients admitted to intensive care units (ICUs) [1]. As a general rule, MDR is defined as acquired non-susceptibility to at least one agent in ≥ 3 antimicrobial categories and XDR is referred as non-susceptibility to at least one agent in all categories but sensitive to ≤ 2 antimicrobial categories [2]. Over the years, selective pressure by administration of different classes of antibiotics has resulted in micro-organisms bearing additional types of resistance mechanisms that led to MDR (enzymatic mechanisms of drug modification, enhanced efflux pump expression, novel penicillin-binding proteins (PBPs), mutated drug targets, and altered membrane permeability) [8]. Bacterial resistance to antibiotics is due to acquired or intrinsic mechanisms [8]. Inadequate surveillance, misuse of antibiotics, and excess administration of antibiotics in the livestock industry resulted in the appearance and spread of MDR/XDR bacteria all over the world [3, 9].

Today, resistance to antibiotics and disinfectants in various bacterial strains is a major public health problem in the world, with increasing growth worldwide. Reports of this have generally been based on the detection of antibiotic resistance in bacteria associated with nosocomial infections, but in recent years with the identification of MDR, strains in different countries, ways to spread and spread the relevant genes are important [4, 5].

One of the most important factors in the spread of nosocomial infections is the improper use of disinfectants. As none of the disinfectants are equally suitable for all different disinfection needs, studies are needed to determine the disinfectant effects of different disinfectants so that you can choose the right disinfectant [5–7].

Improper use of disinfectants, dilution in the environment after discharge, and biodegradation result in biocide concentration gradients. As a result, microorganisms are alternately exposed to non-lethal concentrations of disinfectants. Recent studies have shown that, when bacteria are exposed to non-lethal concentrations of disinfectants, it facilitates resistance to disinfectants and may also lead to resistance to other antimicrobials, such as antibiotics [10–14]. There is a growing concern that the widespread use of disinfectants has also been involved in antibiotic resistance [15, 16].

*P. aeruginosa* have mechanism of developing resistance to disinfectants and antibacterial agents. Among these, small multidrug resistance (SMR) proteins are located in the inner layer of the cytoplasmic membrane and are divided into three groups: SUG, SMP, and PSMR. They cause resistance to biocides and a number of antibiotics. The genes *qacE* and *qacEΔ1* are located in the SMP subgroup and have been identified on plasmids and integrons of many gram-negative and gram-positive bacteria that are resistant to drugs. *qacEΔ1* gene is a mutation of the *qacE* gene [17]. *SUG* genes are also located on the plasmid. The SMR family includes proton-dependent efflux pumps [18]. Qac genes is resulted in resistance against quaternary ammonium compounds, also these genes code for resistance to a broad spectrum of other cationic compounds such as biguanides, diamidines and intercalating dyes [19]. In *P. aeruginosa* the efflux pumps prevent the formation of lethal concentrations of toxic compounds by directing extracellular compounds (antibiotics, chlorhexidine, various drugs, etc.) out of the bacteria, and promotes the survival of the pathogen. Increased expression of efflux pump genes causes MDR isolates [20, 21]. Close association between resistance to antibiotics and biocides can be explained by the fact that a variety of antibiotic resistance genes are harbored by class 1 integrons (mobile genetic elements). Therefore, there is a global concern that the inappropriate use of disinfectants could select gram-negative antibiotic-resistant bacteria [22, 23].

*P. aeruginosa* use biofilm formation as another mechanism to resist disinfectants [24]. One of the purposes of the current study is to determine the correlation between antibiotic/detergent resistance and biofilm formation in *P. aeruginosa*. Treatment of *P. aeruginosa* infections is challenging, due to the acquired and intrinsic resistance of *P. aeruginosa* against a wide range of antibacterial agents [25, 26], biofilm formation, enzyme production suppression, and overexpression of efflux pumps, known as resistance mechanisms of this microorganism [27].

The present study identified resistance and sensitivity to common disinfectants in two steps: with or without Ethylene-diamine-tetra-acetic acid (EDTA). The disinfectants examined in the current study were Sodium hypochlorite 5%, Ethanol alcohol 70%, Sayasept- HP 2%, Chlorhexidine 2%, Dettol 4.8%. Based on the studies conducted in Hospitals in Iran, as well as surrounding countries, disinfectants were selected for this study. These disinfectants are also widely used worldwide for disinfection purposes. Detection of efflux pump genes (*qac-E*, *qacE-Δ1*, and *sug-E1*) by PCR technique was performed. *P. aeruginosa* strains were also evaluated in terms of biofilm production. Also, the relationship between resistance to disinfectant, and biofilm production was assessed. In the present study, the relationship between resistance to disinfectants, and antibiotics was investigated. The effect of exposure to sub-inhibitory concentrations of Sodium hypochlorite on antimicrobial susceptibility test was determined.

## Results

**Fig.1:**
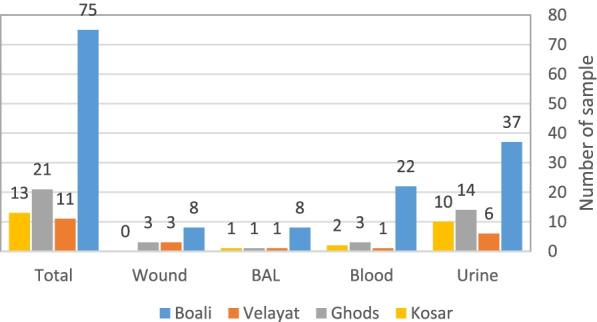
Graph of relative frequency distribution of *P. aeruginosa* isolates, according to the type of clinical isolation from patients and Hospitals

A total of 120 (12.1%) *P. aeruginosa* strains were collected from 986 clinical specimens of Hospitalized patients. Out of 120 obtained isolates, 67 (55.8%) were from Urine, 11 (9.2%) of them from Broncho Alveolar Lavage (BAL), and 28 (23.3%), 14 (11.7%) of them were from blood and wound respectively (Fig. 1). In the current study, all isolates were identified by biochemical tests (Table 1).

**Table 1:** Biochemical test and identification of *P. aeruginosa*

Gram Staining	Negative
Shape (Cocci/Diplococci/Rods)	Rods
Motility (Motile / Non-Motile)	Motile
Catalase	Positive
Oxidase	Positive
MR	Negative
VP	Negative
OF (Oxidative/Fermentative)	Oxidative
Indole	Negative
Citrate	Positive
Urease	Negative
H2S	Negative
Gas	Positive
Pigment	Positive
Cetrimide Test	Positive

### Antimicrobial susceptibilities

**Fig. 2:**
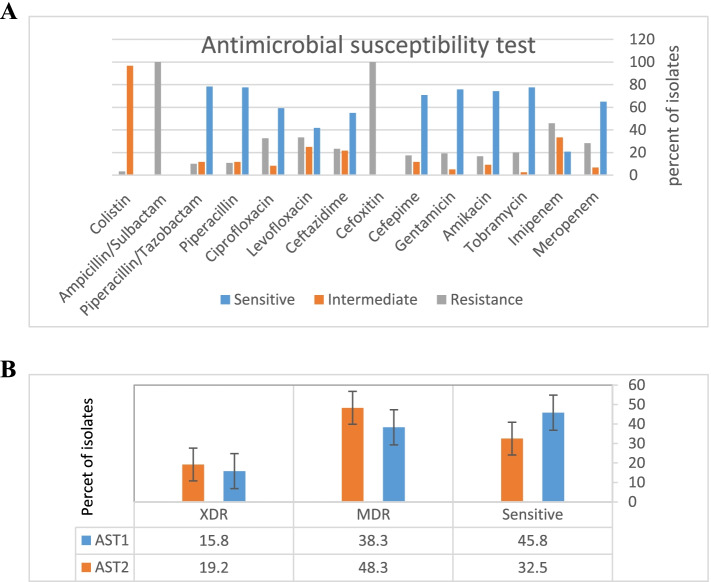
Diagram of the results of antibiotics susceptibility test **A** before exposure to sodium hypochlorite. **B** Comparative diagram of the results of antibiotics susceptibility test before (AST1) and after (AST2) exposure to sodium hypochlorite

The highest resistance rate was against Cefoxitin and Ampicillin/Sulbactam (100%) followed by Imipenem (45.8%) and Levofloxacin (33.3%). The highest susceptibility rate was related to Colistin 116 (96.7%), Piperacillin/Tazobactam 94 (78.3%), Piperacillin 93 (77.5%), Tobramycin 93 (77.5%), and Gentamicin 91 (75.8%) respectively (Table 2 and 3) (Fig. 2A).

**Table 2:** Percentage of antibiotic resistance and susceptibility by each antibiotic

	**AST isolates**	**Sensitive %**	**Intermediate %**	**Resistance %**
Amikacin	Sensitive	45.6	0	0
	MDR	24.9	7.5	5.8
	XDR	3.3	1.7	10.8
Gentamicin	Sensitive	44	0	1.7
	MDR	29	3.3	5.8
	XDR	2.5	1.7	11.6
Tobramycin	Sensitive	45.7	0	0
	MDR	30	1.7	6.6
	XDR	1.7	0.83	1.3
Meropenem	Sensitive	39	0.83	5.8
	MDR	25.7	5	7.5
	XDR	0	0.83	15
Imipenem	Sensitive	12.5	17.4	15.8
	MDR	7.5	15	15.8
	XDR	0.83	0.83	14.1
Ceftazidime	Sensitive	39	5	1.7
	MDR	14	16.6	7.5
	XDR	1.7	0	14.1
Cefepime	Sensitive	44	0	1.7
	MDR	24	10.8	3.3
	XDR	2.5	0.83	12.5
Cefoxitin	Sensitive	0	0	45.8
	MDR	0	0	38.3
	XDR	0	0	15.9
Piperacillin	Sensitive	45.7	0	0
	MDR	25.7	10.8	1.7
	XDR	5.8	0.83	9.1
Piperacillin/Tazobactam	Sensitive	45.7	0	0
	MDR	25.7	10.8	1.7
	XDR	6.6	0.83	8.3
Levofloxacin	Sensitive	31.5	9.1	5
	MDR	8.3	15.8	14.1
	XDR	1.7	0	14.1
Ampicillin/Sulbactam	Sensitive	0	0	45.8
	MDR	0	0	38.3
	XDR	0	0	15.9
Ciprofloxacin	Sensitive	42.3	1.7	1.7
	MDR	15.8	5.8	16.6
	XDR	0.83	0.83	14.1
Colistin	Sensitive	-	45.7	0
	MDR	-	34.9	3.3
	XDR	-	15.8	0

**Table 3:** Antibiotic Susceptibility of clinical *P. aeruginosa* isolates before and after incubation with Sodium hypochlorite

**Antibiotic (**Classes**)**	**Before** n (%)			**After** n (%)			*P*-value
	Sensitive	Intermediate	Resistance	Sensitive	Intermediate	Resistance	
**Meropenem**(Carbapenems)	78(65)	8(6.7)	34(28.3)	65(54.2)	15(12.5)	40(33.3)	< 0.001
**Imipenem** (Carbapenems)	25(20.8)	40(33.3)	55(45.8)	16(13.3)	40(33.3)	64(53.3)	0.004
**Tobramycin**(Aminoglycosides)	93(77.5)	3(2.5)	24(20)	83(69.2)	9(7.5)	28(23.3)	0.002
**Amikacin** (Aminoglycosides)	89(74.2)	11(9.2)	20(16.7)	82(68.3)	12(10)	26(21.7)	0.016
**Gentamicin** (Aminoglycosides)	91(75.8)	6(5)	23(19.2)	81(67.5)	11(9.2)	28(23.3)	0.002
**Cefepime** (Cephalosporins)	85(70.8)	14(11.7)	21(17.5)	76(63.3)	18(15)	26(21.7)	0.004
**Cefoxitin**(Cephalosporins)	0(0)	0(0)	120(100)	0(0)	0(0)	120(100)	-
**Ceftazidime** (Cephalosporins)	66(55)	26(21.7)	28(23.3)	60(50)	28(23.3)	32(26.7)	0.031
**Levofloxacin** (Fluoroquinolones)	50(41.7)	30(25)	40(33.3)	44(36.7)	33(27.5)	43(35.8)	< 0.001
**Ciprofloxacin** (Fluoroquinolones)	71(59.2)	10(8.3)	39(32.5)	59(49.2)	17(14.2)	44(36.7)	< 0.001
**Piperacillin** (Β-Lactam)	93(77.5)	14(11.7)	13(10.8)	93(77.5)	14(11.7)	13(10.8)	1
**Piperacillin/Tazobactam** (Beta-Lactamase Inhibitor)	94(78.3)	14(11.7)	12(10)	94(78.3)	14(11.7)	12(10)	1
**Ampicillin/Sulbactam** (Beta-Lactamase Inhibitor)	0(0)	0(0)	120(100)	0(0)	0(0)	120(100)	-
**Colistin** (Polymyxin B)	-	116(96.7)	4(3.3)	-	116(96.7)	4(3.3)	-

^*^Data were classified into sensitive and insensitive groups for statistical analysis. Intermediate group was considered as resistance

Table 2 shows the percentage of antibiotic resistance and susceptibility by each antibiotic. A comparative table of the results of antibiotics susceptibility tests before and after exposure to Sodium hypochlorite is shown in Table 3. Base on susceptibility testing results, 65(61.7%) and 19(15.8%) isolated strains were categorized as MDR and XDR strain respectively.

The changes in the antibiotic-resistance profiles after exposure to sub-inhibitory concentrations of Sodium hypochlorite were observed for different classes of antibiotics (Table 3). Most of *P. aeruginosa* strains showed increased resistance to different kinds of classes of antibiotics, with respect to resistance Meropenem 13(10.8%), Ciprofloxacin 12 (10%), Tobramycin and Gentamicin 10 (8.3%), Imipenem and Cefepime 9 (7.5%), Amikacin 7 (5.9%), Ceftazidime and Levofloxacin 6 (5%) showed the most changes. As a result, the rate of MDR 16 (15.4%) and XDR 4 (3.4%) increased (Fig. 2B).

### Determination of minimal inhibitory concentration (MIC) and minimal bactericidal concentration (MBC) of disinfectants

**Fig. 3:**
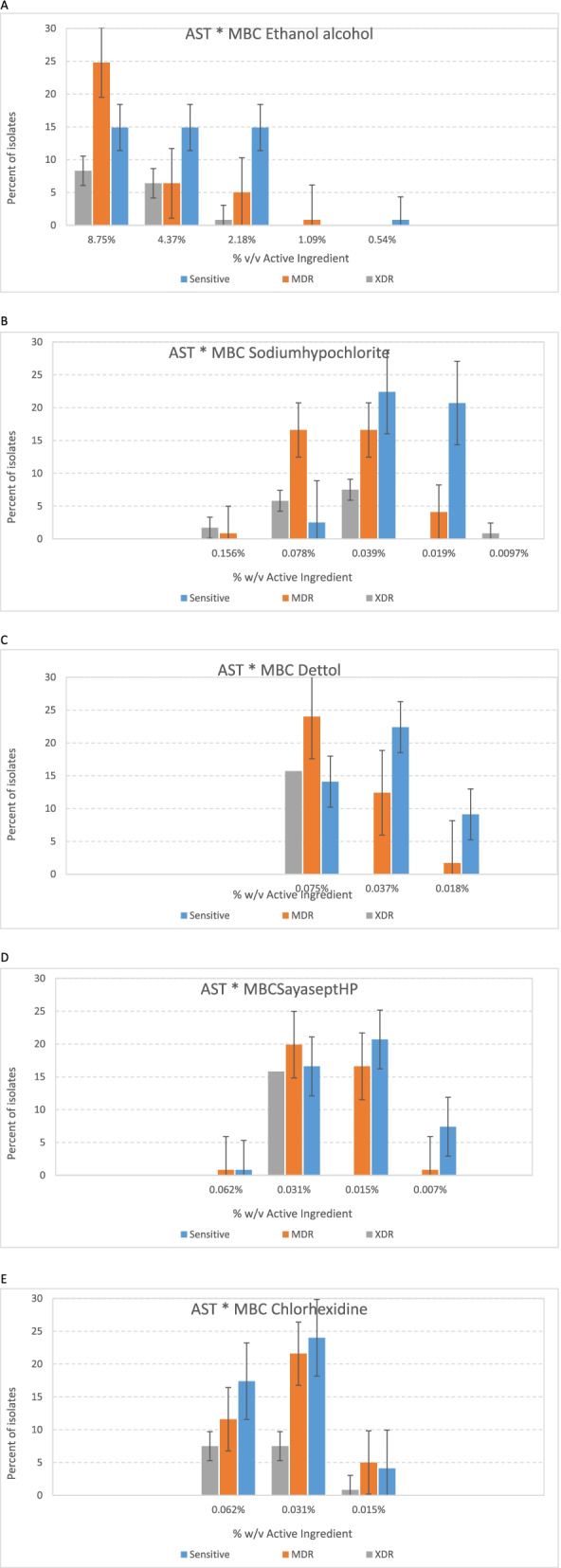
Comparative diagram of the results of antibiotics susceptibility test (MDR/XDR/Sensitive) in different concentration. **A** MBC Ethanol. **B** MBC Sodium hypochlorite. **C** MBC Dettol. **D** MBC Sayasept-HP. **E** MBC Chlorhexidine

In this study the most effective disinfectants were Sodium hypochlorite 5%, Chloroxylenol (Dettol) 4.8%, Sayasept-HP 2%, Chlorhexidine 2%, and Ethanol 70%, respectively. Moreover, the disinfectant susceptibility testing in this study confirmed 1/32 concentration of Sodium hypochlorite and Dettol have lethal effect (MBC) on 120 (100%) of the *P. aeruginosa* isolates. On the other side, the less effective disinfectant was Ethanol 70% and 59(49.2%) of isolates did not show the lethal effect on 4.37% concentration of Ethanol (Table 4). Also results of the current study imply that higher concentration of disinfectants (higher MBC) needs to kill MDR/XDR isolates (Fig. 3 A-E).

**Table 4:** The number (%) of isolates with MICs and MBCs

Disinfectants		N(%)	N(%)	N(%)	N(%)	N(%)	N(%)	N(%)
	Serial dilution →	**1/8**	**1/16**	**1/32**	**1/64**	**1/128**	**1/256**	**1/512**
Ethanol 70%	Active ingredients →	**8.75%**	**4.37%**	**2.18%**	**1.09%**	**0.54%**	**0.27%**	**0.13%**
	MIC	12(10)	65(54.2)	30(25)	11(9.2)	2(1.7)	_	_
	MIC + EDTA	1(0.8)	7(5.8)	32(26.7)	68(56.7)	7(5.8)	4(3.3)	1(0.8)
	MBC	59(49.2)	34(28.3)	25(20.8)	1(0.8)	1(0.8)	_	_
	MBC + EDTA	3(2.5)	25(20.8)	51(42.5)	34(28.3)	6(5)	1(0.8)	_
Sodium hypochlorite 5%	Active ingredients →	**0.62%**	**0.31%**	**0.15%**	**0.078%**	**0.039%**	**0.019%**	**0.0097%**
	MIC	_	_	_	15(12.5)	50(41.7)	30(25)	25(20.8)
	MIC + EDTA	_	_	_	6(5)	12(10)	24(20)	78(65)
	MBC	_	_	_	33(27.5)	56(46.7)	30(25)	1(0.8)
	MBC + EDTA	_	_	_	14(11.7)	24(20)	59(49.2)	23(19.2)
Dettol 4.8%	Active ingredients →	**0.6%**	**0.3%**	**0.15%**	**0.075%**	**0.037%**	**0.018%**	**0.0093%**
	MIC	_	_	_	42(35)	39(32.5)	39(32.5)	_
	MIC + EDTA	_	_	_	5(4.2)	26(21.7)	44(36.7)	45(37.5)
	MBC	_	_	_	65(54.2)	42(35)	13(10.8)	_
	MBC + EDTA	_	_	_	32(26.7)	43(35.8)	37(30.8)	8(6.7)
Sayasept- HP 2%	Active ingredients →	**0.25%**	**0.125%**	**0.062%**	**0.031%**	**0.015%**	**0.0078%**	**0.0039%**
	MIC -HP	_	_	_	50(41.7)	38(31.7)	30(25)	2(1.7)
	MIC + EDTA	_	_	_	13(10.8)	29(24.2)	36(30)	42(35)
	MBC	_	_	2(1.7)	63(52.5)	45(37.5)	10(8.3)	_
	MBC + EDTA	_	_	_	24(20)	47(39.2)	41(34.2)	8(6.7)
Chlorhexidine 2%	Active ingredients →	**0.25%**	**0.125%**	**0.062%**	**0.031%**	**0.015%**	**0.0078%**	**0.0039%**
	MIC	_	_	_	71(59.2)	36(30)	13(10.8)	_
	MIC + EDTA	_	_	_	27(22.5)	33(27.5)	43(35.8)	17(14.2)
	MBC	_	_	44(36.7)	64(53.3)	12(10)	_	_
	MBC + EDTA	_	_	16(13.3)	56(46.7)	48(40)	_	_

The cells (-) means is not MBC/MIC for any of isolates

### Synergistic effect of selected disinfectants and Ethylene-diamine-tetra acetic acid (EDTA)

**Fig. 4:**
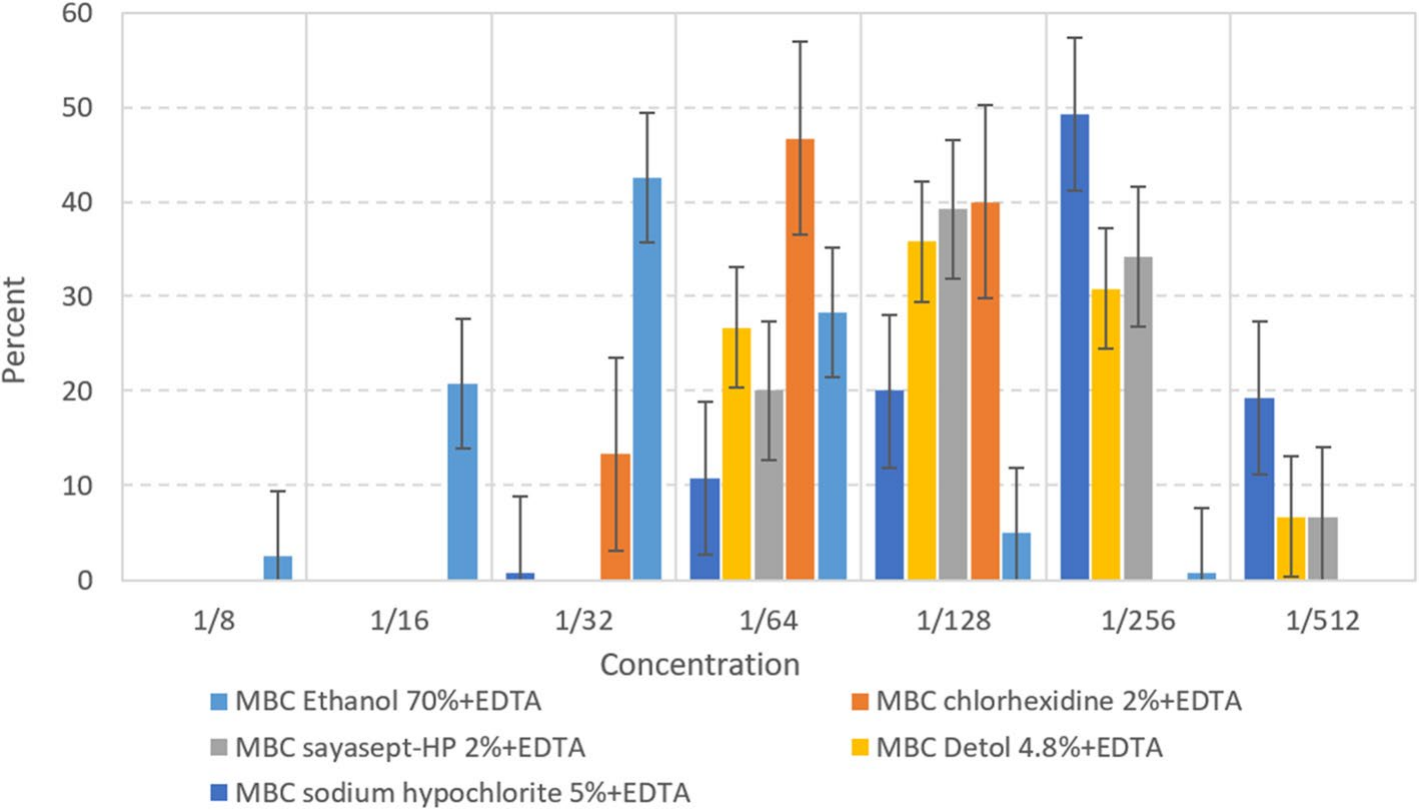
Diagram of MBC ‌disinfectants mixing with EDTA at different concentrations

**Fig. 5:**
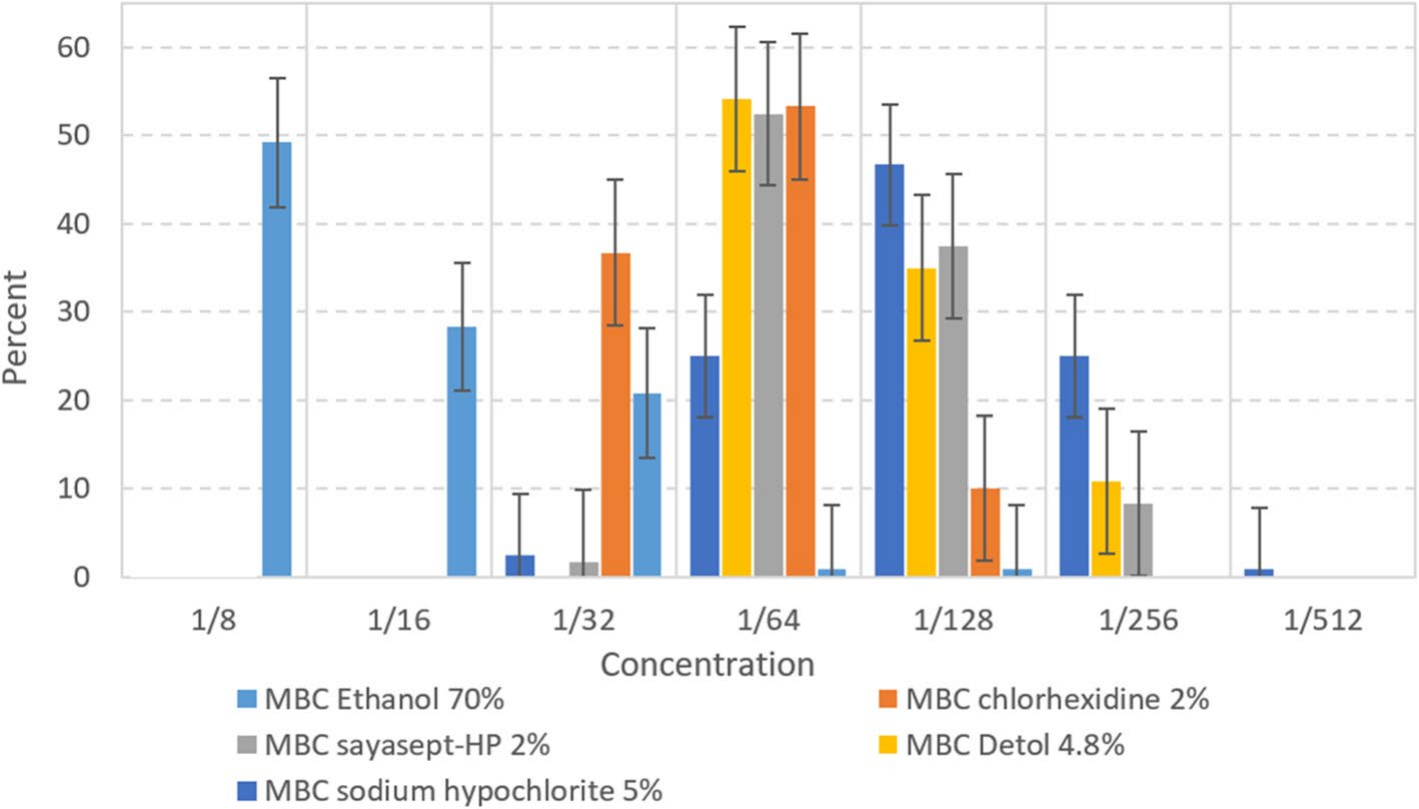
Diagram of MBC ‌disinfectants at different concentrations

Adding EDTA increased the efficiency of all disinfectants included in this study. At the concentrations used, disinfectants without EDTA were efficient at higher concentrations in comparison with mixed EDTA and, when combined with EDTA, a reduction of concentration was observed in MBC and MIC. The effects of EDTA and disinfectants were additive. Ethanol 70%, Sodium hypochlorite 5%, Sayasept-HP 2%, respectively gave the best results when combined with EDTA, although additive effect of EDTA with Chloroxylenol (Dettol) 4.8%, and Chlorhexidine 2% were lower than that of other antiseptics (Table 4). A comparative diagram of MBC ‌disinfectants at different concentrations before and after mixing with EDTA is shown in Figs. 4 and 5.

### Biofilm assessment

**Fig. 6:**
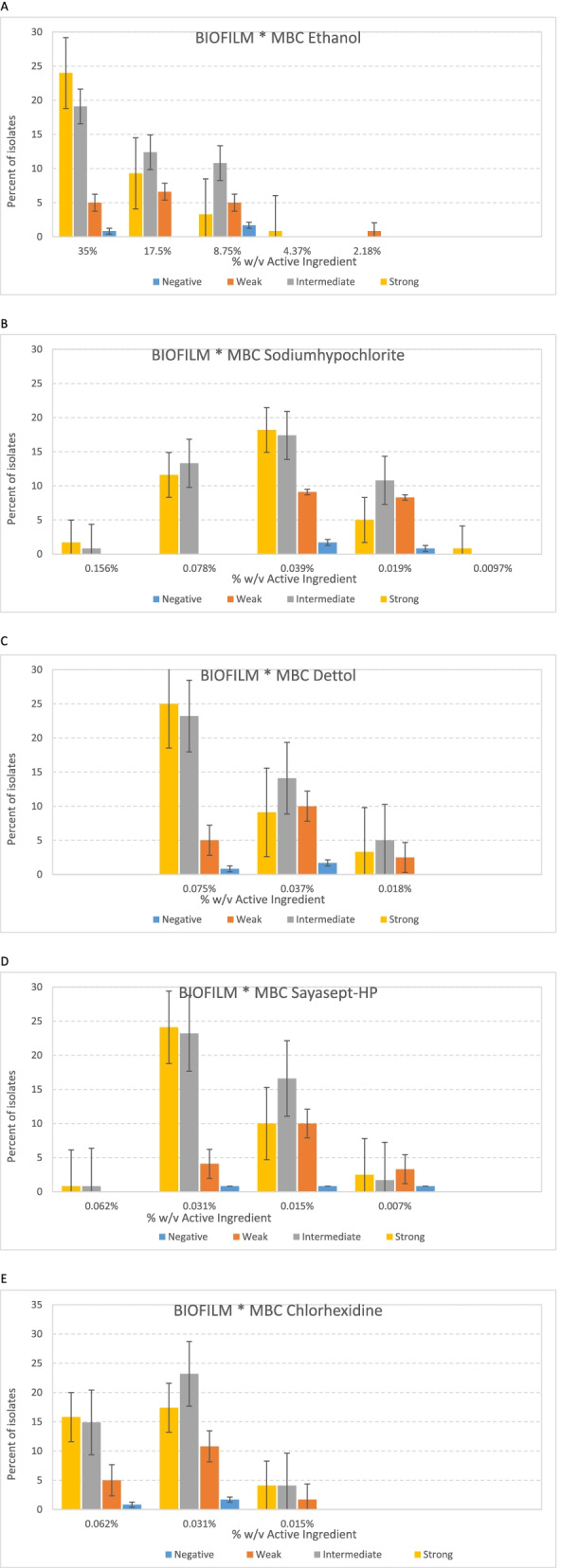
Diagram of the relationship between biofilm formation strength and lethal strength (MBC) of disinfectants at different concentrations. **A** Ethanol alcohol. **B** Sodium hypochlorite. **C** Dettol. **D** Sayasept-HP. **E** Chlorhexidine

In this part, 117 (97.5%) of clinical *P. aeruginosa* isolates were found to develop biofilm, and 45 (37.5%), 51 (42.5%), and 21 (17.5%) isolates were strong, intermediate, and weak biofilm formers respectively, compared to the reference strain. 37 (30.8%) and 28 (23.3%) of strong and intermediate biofilm formers belong to MDR/XDR strains. Also, in the case of strong biofilm-producing isolates, higher concentrations of disinfectants were used to kill the isolates (MBC). Our results indicate, the higher concentrations of biocides (MBC) should be provided to kill strong and moderate biofilm-producing isolates (Fig. 6 A-E).

### Detection of efflux pump genes (qac-E, qacE-Δ1, sug-E1) by PCR technique

**Fig. 7:**
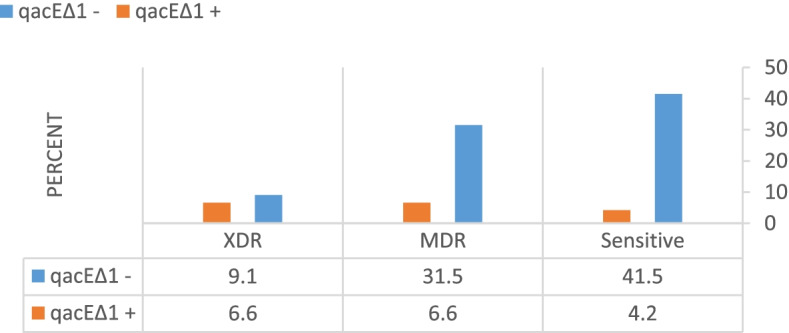
Diagram of gene distribution among sensitive MDR, and XDR isolates

Genomic detection of *qacE*, *qacΔE1*, and *sug-E1* showed that 111 (92.5%), 21 (17.5%), and 114 (95%) out of 120 *P. aeruginosa* isolates harbor the *qacE*, *qacΔE1,* and *sug-E1* genes, respectively. Among the isolates carrying the *qacEΔ1* gene, 16 isolates were MDR / XDR (Fig. 7).

## Discussion

Since 1980, nosocomial infections, caused by *P. aeruginosa* have been classified as a big issue in Hospital, as a result of this problem, medical costs for health care systems have been high [28]. Many studies have shown that disinfectants and antibiotics efficacy is gradually reduced [29]. For this reason, one of the aim of this study was to assess the susceptibility of the isolates to the antibiotics and disinfectants. There are several reasons for the prevalence of disinfectants resistance: inaccurate concentration, inappropriate usage, and insufficient training to prepare and store Hospital disinfectants are among them [30]. Compared to many resistance surveys about antibiotics, the number of global research regarding biocides resistance is insufficient. Due to the clinical importance of *P. aeruginosa*, the efficacy of five Hospital disinfectants was assessed against clinical isolates of *P. aeruginosa*.

The effectiveness of Sodium hypochlorite in the active ingredient concentration of 0.078% at 37 °C for 18–24 h was determined as MBC for all the isolates included in this study, which is more effective between most concentrated active ingredients of different disinfectants in the present study. These results are consistent with another study performed on *P. aeruginosa,* and Sodium hypochlorite 0.5% has been shown to be more effective than Ethanol and Savlone [31]. The results in a study conducted in Brazil showed that Sodium hypochlorite is more effective than ammonium tetravalent compounds against bacteria. In our study, Sodium hypochlorite was more effective than Sayaspet, which is a fifth-generation of ammonium tetravalent compounds [32]. The results of other studies on the active ingredient Sodium hypochlorite confirm the results of our work [33–35]. Also, the results of the current study showed that eradicating of MDR/XDR isolates needs higher concentrations of disinfectants. This highlights the importance of using the suitable disinfectant at the right concentration to kill MDR/XDR bacteria. Given that bacteria are becoming resistant to disinfectants, new disinfectants with new compounds or mixtures must be considered to kill these isolates.

Currently, EDTA has been approved as an antimicrobial agent to reduce the risk of bacterial biofilm formation and colonization. EDTA is known as a metal chelator and disrupts the outer lipopolysaccharide layer of Gram-negative bacteria, and the membrane becomes more penetrable to disinfectants [36]. Another goal of the current study was to determine the synergistic effect of EDTA in combination with five other non-antibiotic antimicrobials. In the current survey, the addition of EDTA increased the efficacy of selected disinfectants significantly. The results showed that disinfectants are able to kill MDR/XDR isolates in lower concentrating with the mixture of the EDTA. A study reported that tetra-sodium EDTA 4% is able to eradicate pre-formed biofilms of clinical isolates [37]. Some surveys revealed that combined antibiotics are more effective compared to single antibiotics [38]. The efficacies of disinfectants currently are being investigated in order to decrease the rate of emerging resistance among clinically isolated bacteria. For decades, EDTA has been known as a potentiating and sensitizing agent. Several studies showed that the action of EDTA biofilm disrupting is due to its ability to cations sequestering (Mg2 + , Ca2 + , and Fe3 +), as a result, increases the effect of other antimicrobial agents [39–41]. The combination of antibiotics and other antimicrobial agents with disodium EDTA has been broadly studied [39, 42, 43]. A study was conducted on Candida and methicillin-resistant *S. aureus* (MRSA), which a combination of Ethanol (25%), EDTA (30 mg/mL), and Minocycline (3 mg/mL) synergistically eradicated pre-formed biofilms [39]. Another survey has been performed on common pathogens involved in canine otitis especially *Pseudomonas,* which revealed that the combination of Tris–EDTA with Chlorhexidine 0.15% has excellent synergistic activity against all isolates [44]. These trials propose that the combination of Chlorhexidine or Ethanol with EDTA does not compromise the activity of one another. However, standard EDTA or disodium EDTA is not a potent and practical antimicrobial agent, even when used at high-level concentrations, and is not able to kill bacteria. On the other side, some studies showed that tetra-sodium EDTA has broad-spectrum antimicrobial activity on its own [37, 41]. It was reported, tetra-sodium EDTA (40 mg/ mL) decreased biofilm colonization by *P. aeruginosa, S. epidermidis, K. pneumoniae, C. albicans,* and *E. coli* on catheter segments [41]. In another survey killing ability of tetra-sodium EDTA 4% against clinically relevant pathogens was reported, and 4% tetra-sodium EDTA kill clinically significant bacterial and fungal pathogens isolated. The tetra-sodium EDTA solution was able to kill all microorganisms tested, at a concentration of 4% or less, and in less than 24 h of exposure [37].

There are studies that are significant to attain a better understanding of the interaction between bacteria and biocides and emerging resistance and cross-resistance in bacteria [45, 46]. In this study, there was a significant difference between the results of the antibiogram before and after exposure to Sodium hypochlorite in most antibiotics. The reason for choosing Sodium hypochlorite to study the effect of sub-inhibitory on *P. aeruginosa* was that it is widely used in most countries in inappropriate concentrations. Our results showed that after exposure to Sodium hypochlorite 16 isolates from antibiotic-sensitive group, categorized as MDR, and among them 4 isolates became XDR. It is worth bearing in mind that, the in-use concentration of disinfectants, in most times is 1000 times greater than of their MIC, to gain a rapid killing of bacteria. Biocide at high-level concentration usually interacts with several targets in the bacterial cell. For this reason, bacteria hardly become resistant via adaptation or other mechanisms. However, bacterial are usually exposed to sub-inhibitory concentrations of biocides. It has been shown that bacteria exposed to sub-inhibitory concentrations of biocides result in increased resistance to biocides and antibiotics in bacteria [12–14, 47]. In a study conducted in 2017, bacteria-harboring biocide resistance genes were more, probable to harbor an antibiotic resistance gene in comparison with bacteria lacking biocide resistance genes [48]. In the current study, we analyzed the correlation between biocides and antibiotics. Positive connections were detected between exposing to the sub-inhibitory concentration of biocides and antibiotic resistance. Such relationships were extensively reported and often involved the up-regulation of efflux pumps [49].

Usage of disinfectants in Hospitals must be intently measured and re-evaluated due to the selection pressure effect of antimicrobials on the advent of resistant bacteria which could be spread to Hospitalized patients. For this reason, resistance is inducible after being exposed to the sub-inhibitory level of disinfectants (sodium hypochlorite), which results in an increase in the isolates resistant to some antibiotics. Therefore, it is noteworthy to evaluate some disinfectants and assess correlations with antibiotic resistance, which should be considered for disinfection practices. It is not correct to consider bacteria that grow in low concentrations of disinfectants as resistant to biocides. This must be determined as ‘increasing MIC value’ or reducing susceptibility, and as a result, it is important to evaluate the bactericidal concentration instead of the inhibitory concentration of disinfectants [50]. It should be noted that the results of different surveys and the methods employed must also be considered. However, opposed results show that there is not any correlation between antibiotic resistance and exposure to sub-lethal concentrations of biocides, which may be due to different bacterial species selected (*Listeria monocytogenes*) or the type of disinfectant selected, which indicates the importance of research on the induction of resistance in different bacterial species with different disinfectants [51]. It is not completely obvious that there is a correlation between biocide resistance and antibiotic resistance, and surveys still continue in this subject [52, 53]. In conclusion, it is clear that biocide concentration is a significant element in bacterial resistance induction. In hospitals, bacteria are exposed to a low concentration of biocide if the disinfectant prepares at low concentrations, and if the diluted disinfectant is kept for a long time [50].

A vital key used by *P. aeruginosa* to survive in harsh environments such as exposure to antibiotics agents is biofilm formation [54]. The National Institute of Heart, Blood, and Lung reported that up to 80% of all infections caused by bacterial are related to biofilm formation [55]. The results of our study showed that 117 (97.5%) isolates formed biofilm, which was similar to other studies [56]. The results of our study revealed that isolates that produced strong and intermediate biofilm are more resistance to antibiotics and disinfectants. Similarly, antibiotic resistance has increased by biofilm formation, resulting in higher antibiotic concentrations in MDR *P. aeruginosa* isolates infections [57]. A study indicated that the rate of *P. aeruginosa* biofilm formation from Iranian patients varied from 48.5% to 99.5%. Generally, the biofilm formation ratio was reported as 87.6%. As well, 27.4%, 30.2%, and 47.7% of *P. aeruginosa* isolates were weak, moderate, and strong biofilm producers, respectively [24]. Accordingly, our data were aligned with the results published in studies where 40–100% of *P. aeruginosa* isolates produce biofilm [58, 59]. Karami et al., reported 73% of both clinical and environmental isolates were biofilm producers [60]. Also, other studies have revealed the importance of biofilm formation by *P. aeruginosa* [61, 62]. In line with our study, it was reported that 58.6% of MDR isolates produce strong biofilm. These results revealed a significant correlation between biofilm formation and MDR isolates [63]. It should be noted that, in contrast to these findings, some studies from different parts of the world indicated a lower prevalence of biofilm formation, and there was no correlation between antibiotic resistance and biofilm-producing [64, 65]. This issue is possibly linked to other resistance mechanisms (efflux pumps, purines, chromosomal mutation, and plasmid acquisition) involved in antibacterial resistance [66].

Another aim of the present study was to investigate the prevalence of *qacE∆1*, *qacE*, and *sug-E1* genes, and their relationship with resistance to antibiotics and biocides in *P. aeruginosa*. In the current study, the prevalence of efflux pump genes was very high, and due to the high prevalence of *qacE* and *sug-E1* genes, no association was found between these genes and resistance to disinfectants and antibiotics. It should be noted that, 21 isolates carrying the *qacE∆1* gene. Between them 16 isolates were MDR or XDR, that indicating an association between this gene and antibiotic resistance. The prevalence of *qacE ∆1* and *sug-E1* genes in our study were consistent with findings of other studies [67–69].

## Conclusions

In the current study, most isolates produced strong and moderate biofilm. The results indicated that strong and moderate biofilm formation isolates need higher concentration (MIC and MBC) of disinfectant for killing. It should be noted that most of MDR/XDR isolates produced strong and moderate biofilm. The present study indicated that EDTA has a significant additive effect in increasing the lethality (MBC) and inhibitory (MIC) power of disinfectants. It is worth noting that EDTA, as a chelating agent of divalent compounds, destroys the biofilm. Therefore, it is suggested to use alternative compounds such as EDTA in combination with disinfectant to increase the potency of disinfectant by creating synergistic effects against MDR/XDR *P. aeruginosa* isolates. EDTA is also fully biodegradable, and has no toxic effects on humans, which is less harmful to the environment and human health than other disinfectants. Collectively, our results revealed that exposure to a sub-inhibitory concentration of Sodium hypochlorite can induce resistance to some antibiotics in *P. aeruginosa*. The results are important because the cross-link between exposure to the Sodium hypochlorite and antibiotic resistance was observed in at least nine antibiotics of the fourteen tested. Appropriate concentration of disinfectants is a critical key to the eradication of bacteria in Hospitals. We also strongly recommend better training on the correct use of disinfectants in Hospitals, since preserving the efficacy of disinfectants is crucial to maintaining hygiene levels in Hospitals and reducing the need for using antimicrobials. This study also showed that Sodium hypochlorite has high lethality and inhibitory power, and Ethanol alcohol has low lethality against isolates of this study. Future studies should include more complex microbial communities residing in the Hospitals, additional *pseudomonas* strains as well as other detergents typically used to clean and disinfect the Hospital surfaces and medical instruments. Based on this study appropriate use of disinfectants at proper concentrations for different species of bacteria should be addressed to avoid inducing resistance mechanisms in bacteria. Also, to the field of study using EDTA in combination with disinfectants should be addressed.

## Materials and methods

### Bacterial strains

A cross-sectional study was performed from April 2019 to July 2020, by approval of the Ethics Committee of Qazvin Medical University (IR.QUMS.REC.1398.156). A total of 120 samples of *P. aeruginosa* were isolated from 986 clinical specimens of Hospitalized patients. Isolates from urine, wound, blood cultures, and BAL were included in this study. All isolates were identified on the basis of cultural, biochemical, and morphological characteristics as per Bergey’s Manual of Systemic Bacteriology [70]. Standard laboratory methods such as growth on Cetrimide agar medium, Triple sugar iron agar, oxidase test, catalase test, Methyl red test, Voges Proskauer test, Citrate utilization, Urease test (Merck, Darmstadt, Germany), Motility test, grow at 42 °C, pigment production was performed to identify *P. aeruginosa* strain. For further investigation, all *P. aeruginosa* isolates cultured in trypticase soy broth (TSB) then were supplemented with 15% glycerol and were stored at − 20 °C. In the next stage, all isolates were confirmed by PCR method. All bacterial culture media were purchased from the Merck, Darmstadt, Germany**.**

### Antibiotic susceptibility testing

Antibiotic susceptibility test (AST) was done using the disk diffusion method, based on the Clinical and Laboratory Standard Institute CLSI 2020 guideline [71] on Mueller–Hinton agar (Merck, Darmstadt, Germany).

Accordingly, a platinum loop was used to pick 3–5 pure *P. aeruginosa* colonies and transferred to a tube containing 5 ml sterile nutrient broth. The isolates were grown in nutrient broth and incubated at 37^0^C until the turbidity was adjusted with the 0.5 McFarland standards (4–6 h). The suspension was evenly swabbed over the surface of Mueller Hinton agar. The inoculated plates were then incubated at 37 °C for 18–24 h. Diameters of the zone of inhibition around the discs were measured, and the isolates were categorized as resistant, intermediate, and sensitive according to the standardized table supplied by the CLSI2020. *P. aeruginosa* ATCC 27,853 was used as control.

Antibiotic disks (MAST Diagnostics, Merseyside, UK) tested were as follows: Piperacillin [30], Piperacillin/Tazobactam (PTZ, 100 μg/10 μg), Ceftazidime (CAZ, 30 μg), Levofloxacin (LEV, 5 μg), Amikacin (AN, 30 μg), Imipenem (IMI, 10 μg), Gentamicin (GM, 10 μg), Meropenem (MEM, 10 μg), Tobramycin (TOB, 10 μg), Ciprofloxacin (CIP, 5 μg), Cefepime (CPM, 30 µg). Cefoxitin (30 μg), Ampicillin/Sulbactam (10 μg /10 μg). *P. aeruginosa* (ATCC 27,853) and *Escherichia coli* (ATCC 25,922) was used as control. The MICs for Colistin were determined using the MIC (micro broth dilution) method, then incubated at 37 °C for 18–24 h. Colistin susceptibility was interpreted according to the CLSI 2020 clinical breakpoints (≥ 4 = resistance, ≤ 2 = intermediate) [71].

To determine the effect of exposure to sub-inhibitory concentrations of Sodium hypochlorite on antimicrobial susceptibility, an antibiogram was done after exposing to sub-inhibitory concentrations of Sodium hypochlorite, and results were compared with before exposing. For this goal, an antibiogram test was performed for bacteria that had grown in the highest concentration of Sodium hypochlorite. The test was performed in the same manner as described for determining the MIC and MBC. Briefly, 20 µL of the suspension in the MIC wells were aseptically transferred to the 5 ml sterile nutrient broth. The isolates were grown in nutrient broth and incubated at 37^0^C until the turbidity was adjusted with the 0.5 McFarland standards (4–6 h). The suspension was evenly swabbed over the surface of Mueller Hinton agar. The inoculated plates were then incubated at 37 °C for 18–24 h. Diameters of the zone of inhibition around the discs were measured, and the isolates were categorized as resistant, intermediate, and sensitive according to the standardized table supplied by the CLSI2020. *P. aeruginosa* ATCC 27,853 was used as control.

### Determination of disinfectants MICs and MBCs

In Iranian Hospitals, Ethanol 70%, Chlorhexidine 2%, Sodium hypochlorite 5%, Chloroxylenol (Dettol) 4.8%, Sayasept- HP 2% are the most applicable disinfectants (Table 5). The MICs and MBCs of the all mentioned disinfectants were assessed by broth micro-dilution method (micro titer assay, 96-well plate) [72]. Briefly, 100 μl nutrient broth was added to all wells. Then 100 μl of disinfectant was added to well one, after serial dilution, 100 μl of bacterial inoculum (10^6^ cfu/ml) was added to all wells. Micro plates were then incubated at 37 °C for 24 h [31, 73, 74]. The dilutions included 1/2, 1/4, 1/8, 1/16, 1/32, 1/64, 1/128, 1/256, and 1/512, Active ingredient of disinfectants are available in Table 6. MICs and MBCs were calculated. wells 11 and 12 are positive (TSB + inoculation) and negative (TSB + antimicrobial) controls [75–77]. The lowest concentration of biocide that inhibits bacterial growth and does not show turbidity is reported as the minimum inhibitory concentration (MIC). Subsequently, 100 μl of four final clear diluted wells of each disinfectant were cultured on Muller Hinton agar medium and if after 48 h at 37° C 99.9% of the bacteria did not grow (i.e. no growth or growth Less than 15 colonies) That dilution is considered the minimum bactericidal concentration (MBC) [78, 79]. Rideal-Walker Phenol Coefficient Test was used to determine the efficacy of the disinfectants (Table 7) [80].

**Table 5:** Main properties of the five disinfectants used in the study

Generic name Working	Manufacturer	Chemical composition
Domestic Bleach	Golrang, Iran	Sodium Hypochlorite (40 G/L)
Chlorhexidine Digluconate	Sigma-Aldrich	20% (W/V) Chlorhexidine Digluconate
Ethanol	Razi, Iran	70% (V/V) Ethanol
Dettol	British Company Reckitt	Chloroxylenol Comprises 4.8% Of Dettol's
Sayasept-HP	Behban Chemistry, Iran	Fifth-Generate Qacs

**Table 6:** Active ingredient of disinfectants based on serial dilution.

Disinfectant	1/2	1/4	1/8	1/16	1/32	1/64	1/128	1/256	1/512
Ethanol alcohol 70%	35%	17.5%	8.75%	4.37%	2.18%	1.09%	0.54%	0.27%	0.136%
Sodium hypochlorite 5%	2.5%	1.25%	0.62%	0.31%	0.156%	0.078%	0.039%	0.019%	0.0097%
Dettol 4.8%	2.4%	1.2%	0.6%	0.3%	0.15%	0.075%	0.037%	0.018%	0.0093%
Sayasept- HP 2%	1%	0.5%	0.25%	0.125%	0.062%	0.031%	0.015%	0.007%	0.0039%
Chlorhexidine 2%	1%	0.5%	0.25%	0.125%	0.062%	0.031%	0.015%	0.007%	0.0039%

**Table 7:**
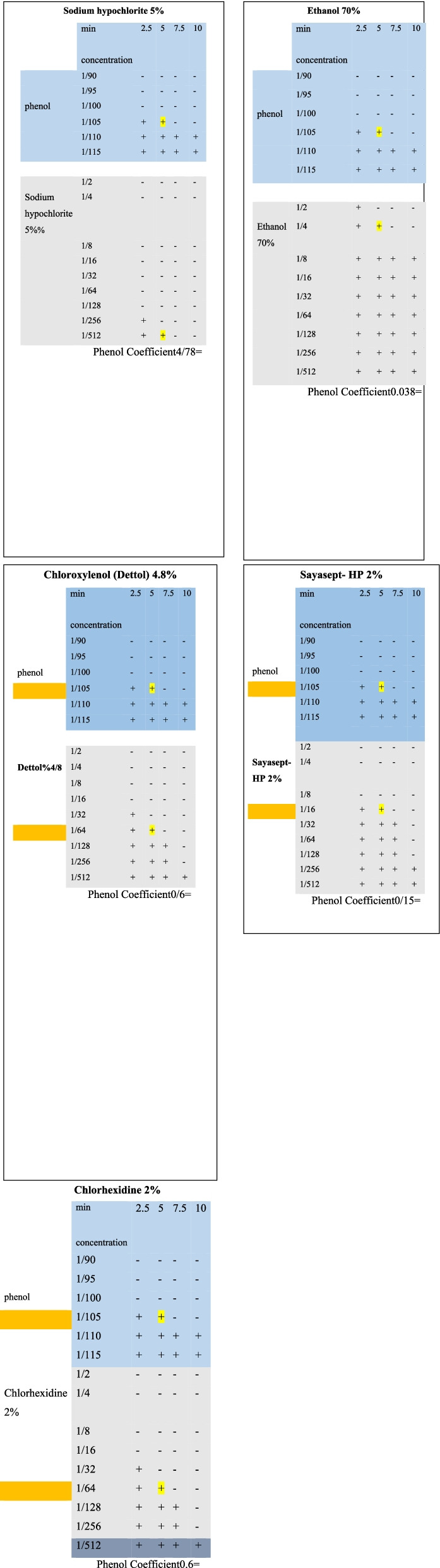
Rideal-Walker Phenol Coefficient test

### Efficacy of Ethylene-diamine-tetra acetic acid (EDTA) on selected biocides

For this purpose, the selected disinfectants were mixed equally with the EDTA 17% and placed at room temperature for 15 min. Then, for all isolates MIC and MBC with a new mixture were calculated. The obtained results were compared with the previous results and its synergistic effect was investigated [39, 43].

### Assessment of biofilm formation capacity

The biofilm-forming ability was determined using the crystal violet staining method in triplicates and repeated three times for each strain, as previously described [81, 82]. *P. aeruginosa* ATCC 27,853 was used as a positive control, and LB medium was used as a negative control [81]. The bacterial isolates were inoculated with turbidity equal to 0.5 McFarland (1.5 × 10^8^ CFU mL^− 1^). A 200-µL suspension was incubated in each well at 35 °C. After 48 h, the wells were washed three times with phosphate buffer. Following incubation with 1% crystal violet dye (200 µL/well) at 25˚C for 20 min, the wells were washed three times with phosphate buffer and dried. Finally, Ethanol 95% (200µL/well) was added, and optical absorbance was measured at 550 nm (Thermo Scientific GmbH, Driesch, Germany). Biofilm formation was classified into four different groups using the following formulas: If OD < ODc, the biofilm was not formed (negative), If ODc < OD < 2xODc, the biofilm was weak, if 2xODc < OD < 4xODc, the biofilm was moderate. If 4xODc < OD, the biofilm was strong (Table 8) [81].

**Table 8:** Values of biofilm formation by bacterial isolates

OD values	Biofilm Formation
<ODc	None
ODc<ODt ≤ 2*ODc	Weak
2*ODc<ODt ≤ 4*ODc	Moderate
4*ODc<ODt	High

### Molecular method for detection of antiseptic resistance gene

Detection of antiseptic resistance gene was performed by polymerase chain reaction (PCR) method; primer sequences used are available in Table 9. Genomic DNA was extracted using the boiling method [83]. Briefly, the isolates were cultured on trypticase soy agar (TSA) and incubated for 20 h at 37 °C. Three colonies were selected and inoculated into 400 μL of Tris–EDTA (TE) buffer, in the next stage heated at boiling temperature (100 °C) for 10 min, and then cooled down on the ice for 15 min. Next, the tube was centrifuged at 11,000 rpm, and the supernatant was used as genomic DNA for the PCR assay. For PCR amplification, each reaction was performed in a final volume of 25 μL containing 12.5 μL of 2 × Taq PCR Master Mix (SinaClon Bioscience Co, Tehran, Iran), 0.5 μL 10 pM of each forward and reverse primer, 3 μL of DNA template, and 8.5 μL Sterile distilled water [83]. The PCR conditions were as follows: pre-denaturation at 94 °C for 5 min, 30 cycles of DNA denaturation for 1 min at 94 °C, annealing based on Table 10, extension for the 50 s at 72 °C, and a final extension at 72 °C for 10 min. The PCR products were electrophoresed on 1% agarose gel with 100 V for 50 min, and stained with DNA safe stain. All primers included in this study designed by AlleleID6. In the next stage all primers were tested in the BLAST program at the NCBI Gene Bank and verified.

**Table 9:** The lists of primers were used in this study

Target gene	Product size (bp)	Primer sequence (5ʹ →3ʹ)	Reference
*qac-E*	206	F: 5ʹ-TGCGTTCCTGGATCTATCTG-3ʹ R: 5ʹ-GACGATGCCAATGCCTTC-3ʹ	In study
*qacE*-*Δ1*	202	F: 5ʹ-TTGTTATCGCAATAGTTG-3ʹ R: 5ʹ-AATGGCTGTAATTATGAC-3ʹ	In study
*sug-E1*	196	F: 5ʹ-CCGTTGGTCTGAAATACAC-3ʹ R: 5ʹ-ATGGATTCGCCGAACAGG-3ʹ	In study
*ZntB*	372	F: 5ʹ-GCCAGTTGCGAGTAGATGTC-3ʹ R:5ʹ-CCGTGGAGTGAACCTGAATC-3ʹ	In study

**Table 10:** Temperature and time of different stages of PCR

Final Extention	Extention	Anneling	Denaturation	Primery	Cycle number	Gene
				denaturation		
10Min/72	50 s / 72	50 s / 53	1Min/94	5Min/94	30	*qac-E*
10Min/72	50 s / 72	45 s / 51	1Min/94	5Min/94	30	*qacE-Δ1*
10Min/72	50 s / 72	50 s / 53	1Min/94	5Min/94	30	*SUG-E1*
10Min/72	50 s / 72	50 s / 57	1Min/94	5Min/94	30	*ZntB*

### Statistical analysis

Descriptive statistics were used to measure the characteristics of the study. Pearson chi-square or Fisher's exact test and McNemar’s test was used to determine significant differences between proportion. *P* values of < 0.05 were considered significant. Statistical analysis was performed by using SPSS version 16.0 statistical software (SPSS Inc., Chicago, IL, USA).

## Data Availability

All data and material are available upon request to correspondence author.

## Abbreviations

MDR: Multidrug-resistant
XDR: Extensively drug-resistant
MIC: Minimum inhibitory concentration
MBC: Minimum bactericidal concentration
WHO: World Health Organization
EDTA: Ethylene-diamine-tetra acetic acid
